# P-1661. Characteristics and Outcomes Associated with Treatment of Asymptomatic Bacteriuria Among Patients with Neurogenic Bladder in a Statewide Hospital Database

**DOI:** 10.1093/ofid/ofae631.1827

**Published:** 2025-01-29

**Authors:** Eva Amenta, Barbara Trautner, Jennifer Horowitz, Elizabeth McLaughlin, Tawny Czilok, Steven J Bernstein, Daniel Nielsen, David Ratz, Ashwin Gupta, Anurag Malani, Tejal N Gandhi, Lindsay A Petty, Scott A Flanders, Valerie Vaughn

**Affiliations:** Baylor College of Medicine, Houston, Texas; Michael E. DeBakey Veterans Affairs Medical Center / Baylor College of Medicine, Houston, Texas; Michigan Medicine, Ann Arbor, Michigan; Michigan Medicine, Ann Arbor, Michigan; Michigan Medicine, Ann Arbor, Michigan; University of Michigan, Ann Arbor, MI; Michigan Medicine, Ann Arbor, Michigan; VA Ann Arbor Health System, Ann Arbor, Michigan; Michigan Medicine, Ann Arbor, Michigan; Trinity Health Michigan, Ann Arbor, Michigan; Michigan Medicine, Ann Arbor, Michigan; University of Michigan, Ann Arbor, MI; University of Michigan/Michigan Medicine, Ann Arbor, Michigan; University of Utah Medical School, Salt Lake City, UT

## Abstract

**Background:**

Inappropriate treatment of asymptomatic bacteriuria (ASB) among hospitalized patients leads to longer length of stay (LOS) without improving outcomes. ASB is common in individuals with neurogenic bladder (NB); however, little is known about the epidemiology, treatment, and outcomes of inpatients with NB and ASB.

**Figure:**
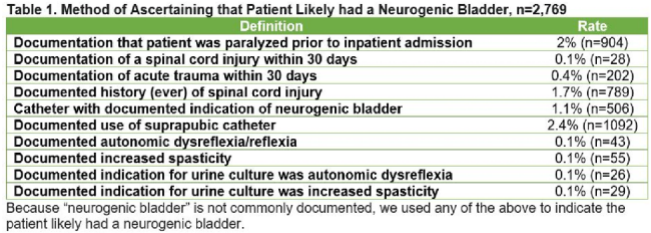

**Methods:**

Between 1/1/2017-12/31/2023, abstractors collected deidentified data—including signs/symptoms of urinary tract infections (UTI) and 30-day outcomes—from inpatients with a positive urine culture (UC) at 46 hospitals. Due to poor documentation of NB, we defined NB using surrogates (**Table 1**). Patients without signs or symptoms of UTI were classified as ASB. Characteristics of patients a) with vs. without NB and b) with NB and ASB who were treated vs. untreated were compared using t or chi-squared tests. Finally, we compared 30-day outcomes of patients with NB and ASB treated vs. untreated using general estimating equation models adjusting for variables known to be associated with the outcomes via inverse probability of treatment weighting.

**Figure:**
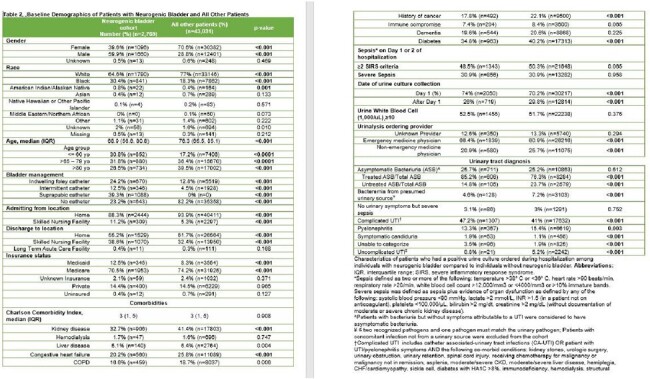

**Results:**

Of 45,800 inpatients with a positive UC, 6% (2,769) had NB (**Table 2**). Compared to patients without NB, patients with NB were more often male (59% vs. 29% p< 0.001), younger (median age 69 vs. 76 p< 0.001), less likely to be white (64.6% vs. 77% p< 0.001) and more likely to use a catheter (76% vs. 17% p< 0.001). While ASB rates (25.7% vs. 25.2%, p=0.62) were similar, bacteremia from a presumed urinary source (4.6% vs. 7.2%, p< 0.001) was less common in patients with NB. 85.1% (605/711) of patients with NB with ASB received antibiotic therapy. Similar to patients without NB, altered mental status (AMS) was the main predictor of ASB treatment (23.8% vs. 13.3% p=0.017; **Table 3**). LOS after UC was longer among those with NB and ASB who were treated vs. untreated (4 vs. 3 days, aIRR: 1.538, 95% CI: 1.33-1.77 p< 0.001; **Table 4)**.

**Figure:**
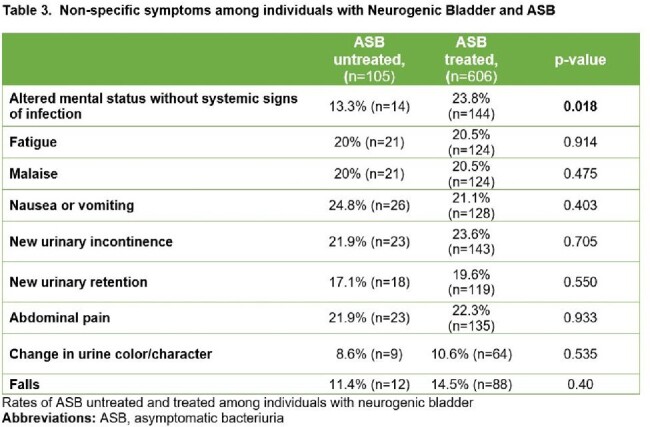

**Conclusion:**

Among inpatients with NB in a statewide dataset, ASB was common, more frequently treated with antibiotics, and treatment was associated with longer LOS. AMS was the main predictor of ASB treatment. Bacteremia was lower among patients with NB, suggesting UC overuse.

**Figure:**
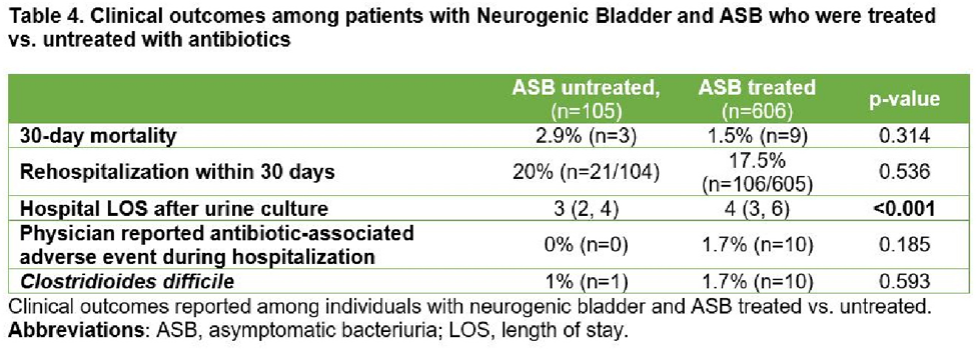

**Disclosures:**

**Barbara Trautner, MD, PhD**, Abbott Laboratories: Stocks/Bonds (Public Company)|AbbVie: Stocks/Bonds (Public Company)|Bristol Myers Squibb: Stocks/Bonds (Public Company)|Pfizer: Stocks/Bonds (Public Company)|Phiogen Pharma: Advisor/Consultant **Elizabeth McLaughlin, MS, RN**, Blue Cross Blue Shield of Michigan: Salary Support

